# Statistical shape modelling of the first carpometacarpal joint reveals high variation in morphology

**DOI:** 10.1007/s10237-019-01257-8

**Published:** 2019-11-21

**Authors:** Wan M. R. Rusli, Angela E. Kedgley

**Affiliations:** Department of Bioengineering, Imperial College London, London, SW7 2AZ UK

**Keywords:** Statistical shape modelling, First carpometacarpal joint, Morphology, Articular tilt angle, First metacarpal torsion angle

## Abstract

The first carpometacarpal (CMC) joint, located at the base of the thumb and formed by the junction between the first metacarpal and trapezium, is a common site for osteoarthritis of the hand. The shape of both the first metacarpal and trapezium contributes to the intrinsic bony stability of the joint, and variability in the morphology of both these bones can affect the joint’s function. The objectives of this study were to quantify the morphological variation in the complete metacarpal and trapezium and determine any correlation between anatomical features of these two components of the first CMC joint. A multi-object statistical shape modelling pipeline, consisting of scaling, hierarchical rigid registration, non-rigid registration and projection pursuit principal component analysis, was implemented. Four anatomical measures were quantified from the shape model, namely the first metacarpal articular tilt and torsion angles and the trapezium length and width. Variations in the first metacarpal articular tilt angle (− 6.3° < *θ* < 12.3°) and trapezium width (10.28 mm < ${\fancyscript{w}}$ < 11.13 mm) were identified in the first principal component. In the second principal component, variations in the first metacarpal torsion angle (0.2° < *α* < 14.2°), first metacarpal articular tilt angle (1.0° < *θ* < 6.4°) and trapezium length (12.25 mm < $\text{ }\ell$ < 17.33 mm) were determined. Due to their implications for joint stability, the first metacarpal articular tilt angle and trapezium width may be important anatomical features which could be used to advance early detection and treatment of first CMC joint osteoarthritis.

## Introduction

In most cases, the thumb works together with the fingers in performing hand-related activities of daily living, for example grip and pinch. Joint disease, such as osteoarthritis at the base of the thumb, in the first carpometacarpal (CMC) joint, will lead to impairment and increase one’s dependency on others in performing daily activities. The first CMC joint is formed by the junction between the first metacarpal and trapezium bones. The biconcave–convex saddle shape of this joint facilitates the wide range of motion of the thumb (Neumann and Bielefeld 2003), but the shape provides little bony stability to the joint (Ladd et al. 2013). The morphology of this joint has been considered as one of the potential factors that contribute to the development of first CMC joint osteoarthritis (Ladd et al. 2014).

Early work by Ateshian et al. (1992) looked at the curvature characteristics and congruence of the articulating surfaces of the first CMC joint, revealing differences between men and women in the curvature of the articulating surface of the trapezium, but not the first metacarpal. This aligned with an even earlier study that reported that women’s first CMC joints are less congruent than men’s (Xu et al. 1998). However, recent studies have not been in agreement, showing no morphological differences in the shape of the articulating surfaces between men and women (Halilaj et al. 2014; Marzke et al. 2012). Up to this point, morphological analyses have been focused on the articulating surface rather than the entire structure of both the first metacarpal and trapezium. It is important not just to analyse regional morphological patterns but also global morphological patterns, and this can be carried out using statistical shape modelling (SSM) (Young and Frangi 2009).

SSM has been used to quantify morphological variability across populations (Bischoff et al. 2014), and this technique allows for both non-metric and metric observations (Cootes et al. 1995). Analysis of the entire structure of the first CMC joint using SSM has determined that men and women have similar morphology (Schneider et al. 2015). Previous studies of the first CMC joint using SSM generally have focused only on non-metric observations (Schneider et al. 2015, 2018), the exception being volume quantification of the first metacarpal and trapezium mean models by Schneider et al. (2015). Anatomical measurement (metric observation) of individual parameters, such as first metacarpal articular tilt angle, has not been carried out, although this anatomical feature has been cited as a risk factor for the development of first CMC joint osteoarthritis (Kim et al. 2016; Kurosawa et al. 2013; Miura et al. 2004). Other parameters, such as the width of the trapezium measured in the Robert’s view radiograph, have also been used to determine thumb osteoarthritis indices (Ladd et al. 2015; Ladd 2018). Another anatomical feature of the first metacarpal that has been quantified previously is torsion (Singh 1979). Difficulty in measuring torsion from x-ray images has limited analysis of this parameter; however, three-dimensional (3D) shape models constructed by SSM of the entire structure of the joint allow for objective measurement. This highlights one utility of SSM for measurement of the variability of these anatomical features across a population.

The purpose of this study was to quantify the morphological variability of the complete structures that comprise the first CMC joint using both multi-object SSM and metric observations, thus enabling further determination of the correlation between the variability in anatomical features of the first metacarpal and trapezium. The hypothesis was that within-bone and between-bone correlations exist between the metric anatomical features of the first metacarpal and trapezium.

## Materials and methods

### Imaging

Computed tomography (CT) images (slice thickness of 1 mm, pixel size of 0.525 mm and resolution of 512 px × 512 px) of 50 cadaveric specimens (mean age = 53.5 ± 10.0 years, 26 right and 24 left hands), consisting of 32 males and 18 females, were used in this study. To determine the variation in the first CMC joint, with an alpha = 0.05 and power of 0.80, a sample size of 31 was necessary; thus, the 50 samples used in this study were adequate to achieve the objective. All CT scans were examined to ensure no osteophytes were present. Ethical approval for the use of these CT images was obtained from the Tissue Management Committee of the Imperial College Healthcare Tissue Bank according to the Human Tissue Act. All specimens used in this study were donated with written informed consent for use in medical research.

Semi-automatic segmentation was carried out on all the CT images using MIMICS (v.17, Materialise, Belgium) to obtain 3D models of the first metacarpal and trapezium. The first metacarpals and trapeziums from left hands were mirrored to ensure all the 3D models had the same coordinate system.

### Statistical shape modelling

**Fig. 1:**
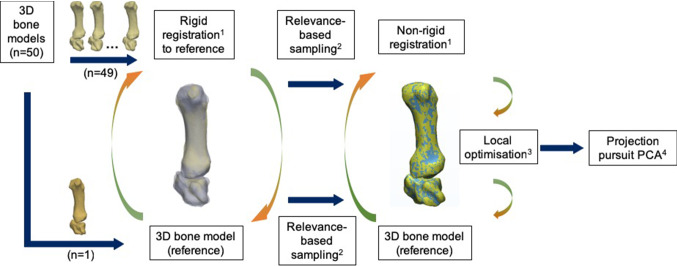
Multi-object SSM pipeline used in this study to obtain the mean model of the first CMC joint and its variations across all principal components. **1** Myronenko and Song 2010; **2** Rodola et al. 2015; **3** Li et al. 2008; **4** Croux et al. 2007

Briefly, the multi-object SSM pipeline consisted of three main processes (Fig. 1). The first process involved the alignment of the source object with the target object. Target object is the term used to represent the 3D bone model that was selected as the reference object, while source object refers to all the other 3D bone models available in the dataset. This was followed by coarse to fine non-rigid registration and finally dimension reduction.

Scaling and alignment of the 3D bone models were carried out using the rigid coherent point drift (CPD) algorithm (Myronenko and Song 2010). Following this, all samples underwent sub-sampling using the relevance-based sampling algorithm (Rodola et al. 2015). The sub-sampling process was carried out to facilitate the coarse to fine non-rigid registration process. The coarse non-rigid registration process was carried out based on the non-rigid CPD algorithm (Myronenko and Song 2010), using the sub-sampled dataset to obtain the initial point-to-point correspondences. The accuracy of the non-rigid registration process was further improved by implementing fine non-rigid registration on the original samples, based on a local optimisation algorithm proposed by Li et al. (2008). By using the original 3D samples during the local optimisation, the effect of sub-sampling during the coarse non-rigid registration process was eliminated. Dimension reduction was carried out using projection pursuit principal component analysis (ppPCA) (Croux et al. 2007) to determine the strongest morphological variations in the dataset used in this study. ppPCA was chosen due to the high dimensionality of the problem. The number of principal components obtained was limited to *n* − 1, where *n* was the total number of 3D bone models in the dataset (Bredbenner et al. 2014). As proposed by Van De Giessen et al. (2009), only principal components that represented more than 5% of the morphological variation were analysed. Leave-one-out analysis was carried out to evaluate the generalisability of the developed statistical shape model.

### Shape analysis

**Fig. 2:**
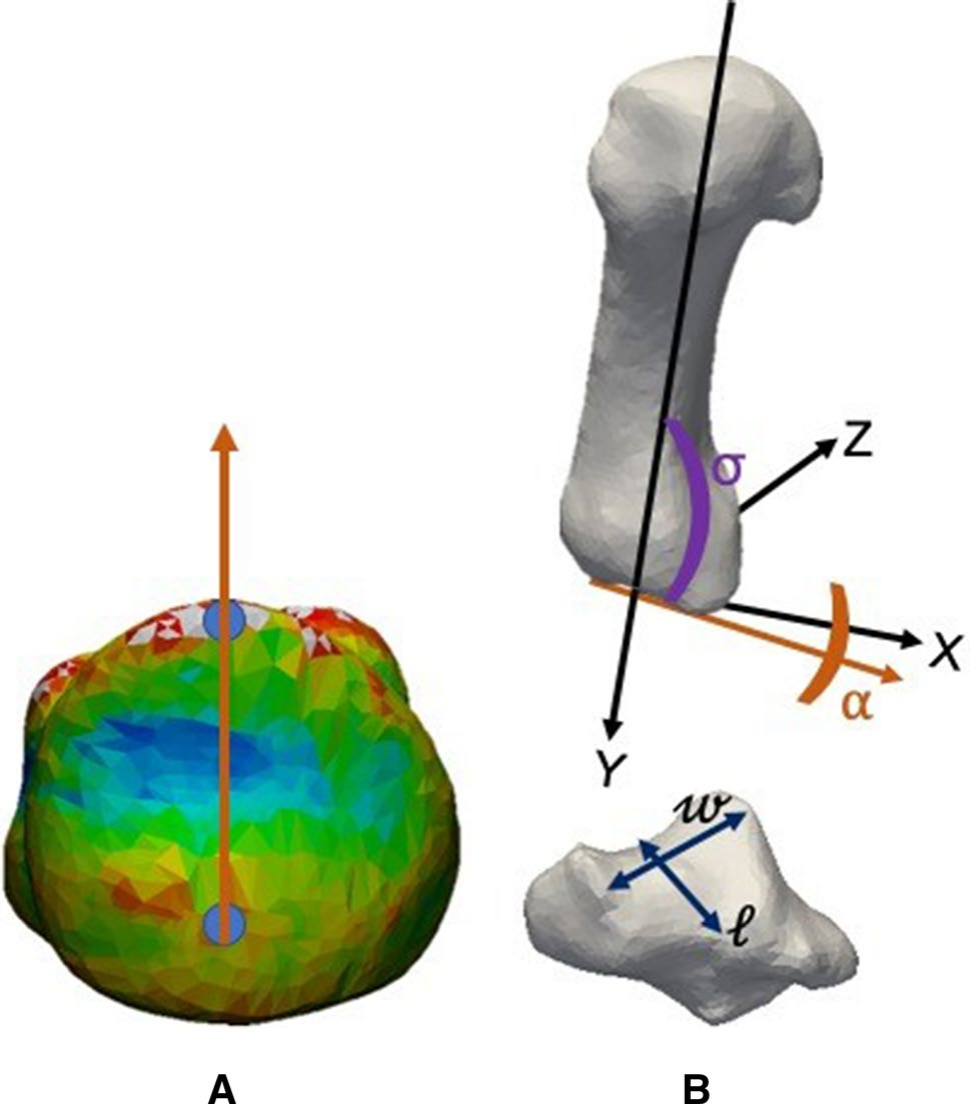
**a** Dorsal and volar cusps of the articulating surface on the first metacarpal were connected by a line (orange). **b** Angle between the y-axis and proximal articular surface line (orange) of the first metacarpal ($\sigma$), first metacarpal torsion angle ($\alpha$) and trapezium length ($\ell$) and width (${\fancyscript{w}}$)

Prior to the shape analysis, the segmental coordinate systems of both the first metacarpal and trapezium were built for the mean shape and its ± 3 standard deviation (± 3SD) bone models based on the recommendations from the International Society of Biomechanics (Wu et al. 2005) in 3-matic software (ver. 9, Materialise, Belgium). Anatomical measurements were performed using 3-matic on the mean shape and ± 3SD of the principal components to determine how the anatomical features of the first CMC joint varied in the population. Two anatomical features of the first metacarpal were measured; these were the first metacarpal articular tilt angle ($\theta$) and the first metacarpal torsion angle ($\alpha$). To provide a reference, a line was constructed based on the volar and dorsal cusps of the proximal articular surface of the first metacarpal (Fig. 2a). For the trapezium, length ($\ell$) and width (${\fancyscript{w}}$) of the first metacarpal facet were measured.

#### First metacarpal articular tilt angle ($\theta$)

$\theta$ was defined as the complementary angle between the tangent to the dorsal cortex and the articular surface of the first metacarpal base, as observed in the lateral view (Kurosawa et al. 2013). Although the dorsal cortex line tangent is relatively easy to construct on an x-ray image, it is not as straightforward to create from a 3D bone model. Therefore, the y-axis of the first metacarpal was used. Finally, $\theta$ was calculated using:

$$\theta = 90^\circ - \sigma$$

where $\sigma$ (Fig. 2b) is the angle between the y-axis and the line created on the proximal articular surface of the first metacarpal.

#### First metacarpal torsion angle ($\alpha$)

Previously, when creating these axes on cadaveric bones, $\alpha$ was defined as the angle between the dorsovolar axes of the first metacarpal head and the articulating surface on the coronal plane (Drapeau 2015; Singh 1979). Figure 2b shows the two axes on the first metacarpal used to determine the $\alpha$. The midpoints of the volar and dorsal sides of the first metacarpal head were used to construct the dorsovolar axis (Drapeau 2015; Singh 1979). However, the x-axis of the first metacarpal and the dorsovolar axis of the first metacarpal head have the same definition, which is the line that divides the first metacarpal in half. This was difficult to locate on the 3D bone models; hence, the x-axis of the first metacarpal was taken to replace the dorsovolar axis of the first metacarpal head. A positive value of first metacarpal torsion represents an ulnar twist of the first metacarpal head relative to the base, while a negative value represents a radial twist of the first metacarpal.

#### Trapezium length, $\ell$, and width, ${\fancyscript{w}}$

$\ell$ and ${\fancyscript{w}}$ were defined as the length and width of the first metacarpal facet on the trapezium, as shown in Fig. 2. The measurement of $\ell$ and ${\fancyscript{w}}$ for the mean shape and ± 3SD of the first metacarpal and trapezium for the first and second principal components were performed using 3-matic.

### Statistical analysis

The measured variability of the anatomical features in the first and second principal components was tested using regression analysis. This was carried out to determine the association of each of the anatomical features with the first and second principal components. The analysis allowed for the identification of any trends in each anatomical feature associated with these components. Pearson’s correlation tests were used to quantify any correlations between the anatomical features of the first metacarpal and trapezium. Only anatomical features that were associated with the first and second principal components, as determined by the regression analysis, were selected. In both statistical tests, significance was assessed as *p* < 0.05 using IBM SPSS Statistics (ver. 25, IBM Corp., Armonk, USA).

## Results

### Statistical shape model of the first CMC joint

**Fig. 3:**
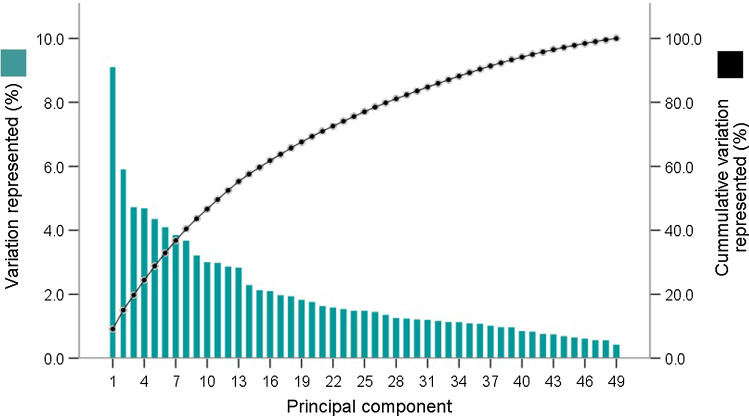
Separate and cumulative variation in morphology represented with the addition of each of the 49 principle components. The first and second principal components represent the greatest proportions of the morphological variation observed in the first CMC joint

The morphological variations in the entire structure of the first CMC joint were represented by 49 principal components (Fig. 3). Resultant multi-object SSM of the entire structure of the first CMC joint shows that the first thirty-six principal components represent 90.3% of the total morphological variation in the population used for this study. The first and second principal components represent 9.1% and 5.9% of the total variation, respectively. The root-mean-squared error obtained from the leave-one-out analysis was 0.5 mm.

**Fig. 4:**
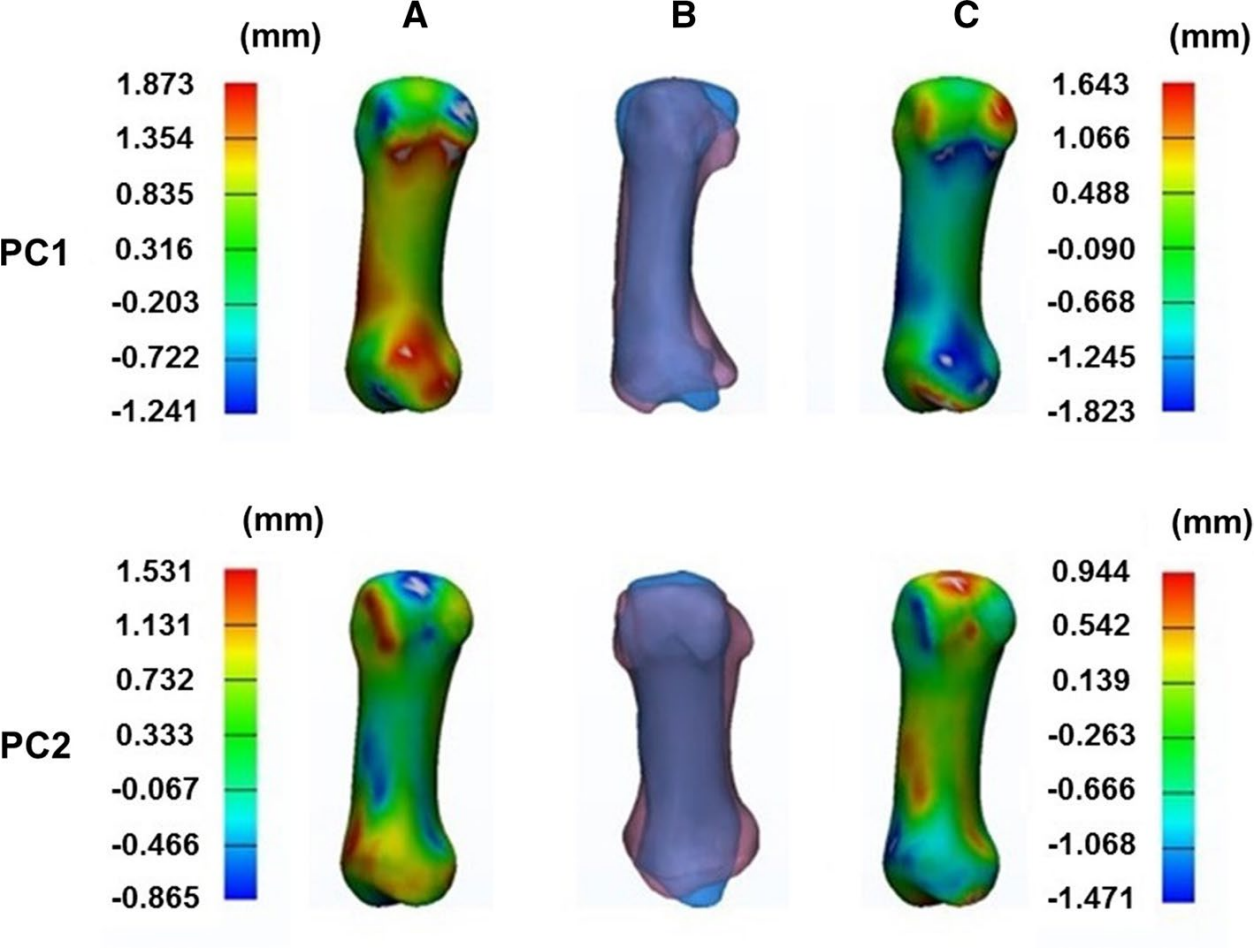
Morphological variations in the first metacarpal in the first (row PC1) and second (row PC2) principal components, represented by distance maps between − 3SD and the mean model (column A) and + 3SD and the mean model (column C), and shapes of the first metacarpal at + 3SD (column B pink) and − 3SD (column B blue)

**Fig. 5:**
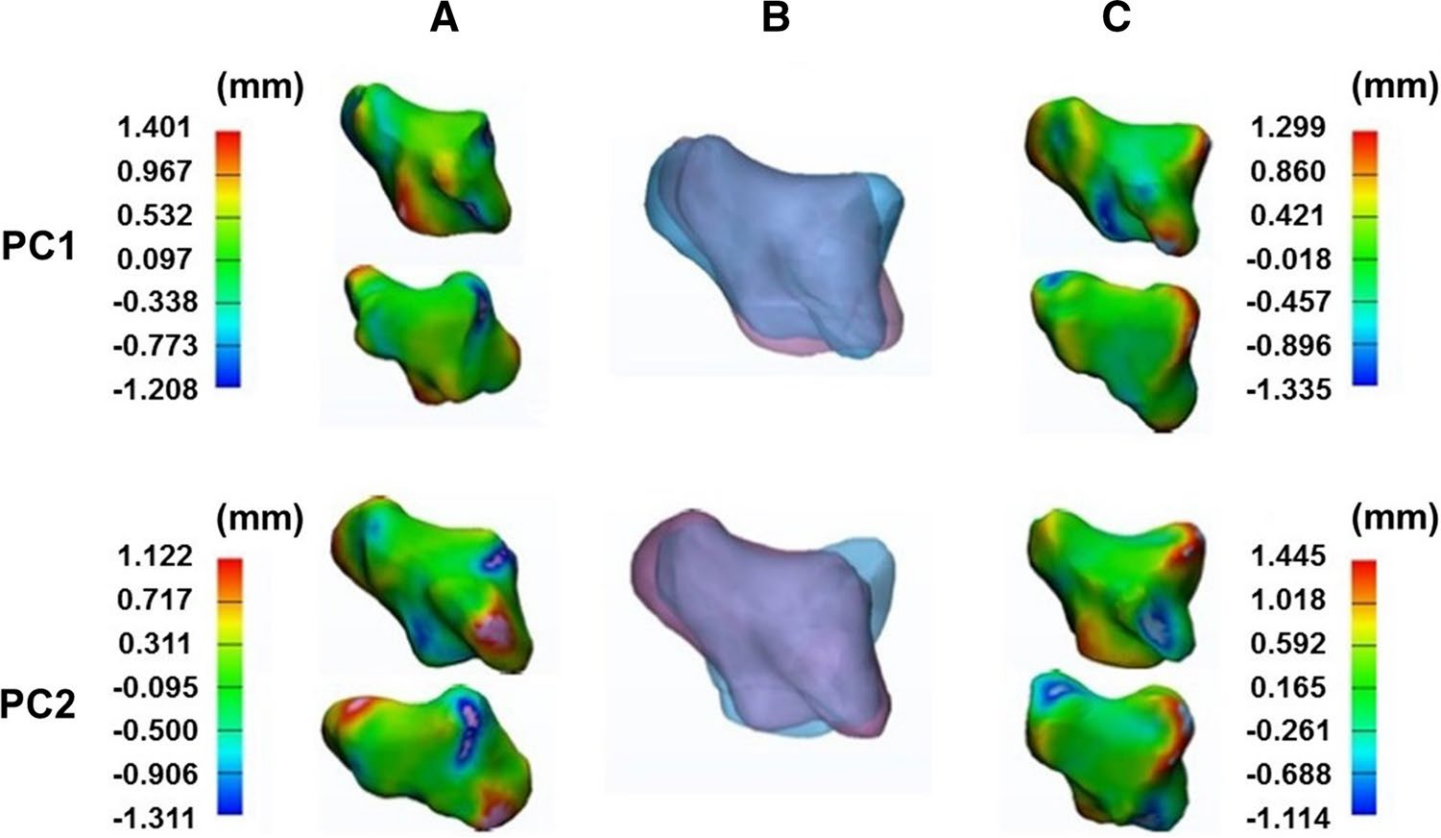
Morphological variations in the trapezium in the first (row PC1) and second (row PC2) principal components, represented by distance maps between − 3SD and the mean model (column A) and + 3SD and the mean model (column C), and shapes of the trapezium at +3SD (column B pink) and − 3SD (column B blue)

Figure 4 shows the morphological variations in the first metacarpal for the first and second principal components determined by multi-object SSM. In the first principal component, there was variation at the proximal end of the first metacarpal, especially in the region of the volar beak. Morphological variations were also observed in the dorsoradial and ulnar regions of the first metacarpal shaft. Both the dorsal and proximal articulating surfaces varied in the second principal component. Variation was also observed in terms of the width at the proximal end of the first metacarpal shaft. The morphological variations in the trapezium for the first and second principal components, as determined by the multi-object SSM, are shown in Fig. 5. In both the first and second principal components, the radial region of the first metacarpal facet on the trapezium was observed to vary the most. In addition, variation was observed in the ulnar region on this articulating surface.

### Anatomical features of the first metacarpal

The articular tilt and torsion angles for the mean model of the first metacarpal were 1.5° and 96.3°, respectively. Table 1 shows the articular tilt and torsion angles for the first and second principal components across ± 3SD. In the first principal component, the articular tilt angle of the first metacarpal ranged between − 6.3° and 12.3°. The regression analysis indicated that the variability in the first metacarpal articular tilt was strongly associated with the first principal component ($R^{2}$ = 0.991, *p* = 0.001). Unlike articular tilt, variability of first metacarpal torsion (− 6.8° < $\alpha$ < 12.7°) was not associated with the first principal component ($R^{2}$ = 0.638, *p* = 0.031). In contrast, first metacarpal torsion variation (0.2° < $\alpha$ < 14.2°) across the population (± 3SD) was strongly associated with the second principal component ($R^{2}$ = 0.91, *p* = 0.01). The first metacarpal articular tilt was similarly associated with the second principal component (1.0° < $\theta$ < 6.4°, $R^{2}$ = 0.829, *p* = 0.04).

**Table 1:** Articular tilt and torsion of the first metacarpal for the first and second principal components across the population used in this study

Weighting coefficient	Principal component 1		Principal component 2	
	Articular tilt $\theta \left(^\circ \right)$	Torsion $\alpha \left(^\circ \right)$	Articular tilt $\theta \left(^\circ \right)$	Torsion $\alpha \left(^\circ \right)$
+ 3SD	12.3	12.7	6.4	0.2
+ 2SD	9.3	7.1	6.5	2.7
+ 1SD	6.0	6.9	3.7	3.6
Mean	1.5	6.3	1.5	6.3
− 1SD	− 1.4	6.3	2.6	6.1
− 2SD	− 4.1	6.1	1.4	8.7
− 3SD	− 6.3	− 6.8	1.0	14.2

### Anatomical features of the trapezium

Table 2 shows the measured values of the trapezium length ($\ell$) and width (${\fancyscript{w}}$) across the population (mean ± 3SD). The length on the mean model of the trapezium was 14.5 mm, and it varied between 14.5 mm and 16.5 mm in the first principal component. In the second principal component, $\ell$ varied between 12.3 mm and 17.3 mm. The regression analysis indicated that $\ell$ was more associated with the second principal component ($R^{2}$ = 0.972, *p* = 0.001) than the first principal component ($R^{2}$ = 0.754, *p* = 0.011). The trapezium width on the mean model was 10.8 mm. In the first principal component, ${\fancyscript{w}}$ varied between 10.3 mm and 11.1 mm across the population (± 3SD; $R^{2}$ = 0.843, *p* = 0.004). However, this anatomical feature was not associated with the second principal component ($R^{2}$ = 0.04, *p* = 0.896).

**Table 2:** Values of the first metacarpal facet length and width on trapezium for the first and second principal components across the population used in this study

Weighting coefficient	Principal component 1		Principal component 2	
	Length $\ell$ (mm)	Width ${\fancyscript{w}}$ (mm)	Length $\ell$ (mm)	Width ${\fancyscript{w}}$ (mm)
+ 3SD	16.5	11.1	17.3	11.9
+ 2SD	16.0	11.0	17.2	10.5
+ 1SD	15.6	11.1	15.6	10.8
Mean	14.5	10.8	14.5	10.8
− 1SD	15.2	10.9	13.5	10.7
− 2SD	14.8	10.4	13.2	10.8
− 3SD	14.6	10.3	12.3	11.6

### Correlation between anatomical features in the first metacarpal and trapezium

Variation in the first metacarpal articular tilt angle was positively correlated with variation in the width of the trapezium ($r$ = 0.902, *p* = 0.005). In the second principal component, the first metacarpal torsion angle was negatively correlated with trapezium length ($r$ = − 0.923, *p* = 0.003). The same strong, but positive, correlation was determined between the first metacarpal articular tilt angle and trapezium length ($r$ = 0.943, i = 0.001). The first metacarpal articular tilt angle had a negative but strong correlation with the first metacarpal torsion angle ($r$ = − 0.847, *p* = 0.016) in the second principal component.

## Discussion

Shape analysis of the first CMC joint, based on multi-object SSM, was successfully performed. Rather than quantifying the morphological variations in the first CMC joint articulating surface alone, multi-object SSM was able to quantify the morphological variations in the entire structure of the first metacarpal and trapezium that form this joint. Only the first and second principal components were analysed (variation in each represented more than 5% of the total; van de Giessen et al. 2010). The remaining principal components were not analysed because the morphological variations observed were very subtle (each represented less than 5% of the total).

Within ± 3SD of the first principal component, the articular tilt angle of the first metacarpal had a range of 18.6°, suggesting that this parameter varies greatly within the population. The first metacarpal articular tilt angle has been found to be higher in first CMC joint osteoarthritis patients, as compared to participants with healthy joints (Kurosawa et al. 2013; Miura et al. 2004). Miura et al. (2004) have suggested that an increase in the first metacarpal articular tilt angle could increase the shear force that translates the first metacarpal dorsally and that this translation can be exacerbated by weakening of the ligaments surrounding the joint. The first metacarpal articular tilt angle also has been found to correlate with the first CMC joint subluxation angle (Kurosawa et al. 2013). While a small articular tilt angle may indicate high proximal first metacarpal facet curvature, this could cause the volar beak of the first metacarpal to abut with the convex surface of the trapezium facet (Marzke et al. 2012). Further, the width of the trapezium has been shown to be a good indicator for the progression of first CMC joint osteoarthritis (Ladd et al. 2015). The strong association between the first metacarpal articular tilt angle and width of the trapezium in the first principal component suggests that perhaps these anatomical features are linked and can be used together as anatomical biomarkers for predicting first CMC joint osteoarthritis.

The size of the trapezoid affects the orientation of the trapezium, positioning the dorsovolar first metacarpal facet towards the radioulnar axis of the other metacarpals (Drapeau et al. 2005; Tocheri et al. 2005). However, this repositioning of the first metacarpal does not facilitate opposition of the thumb (Drapeau 2015). First metacarpal torsion is important in compensating for the orientation of the trapezoid–trapezium–first metacarpal complex to enable the manipulative tasks involved in activities of daily living. In this study, first metacarpal torsion had a range of 19.5° and 14° for ± 3SD of the first and second principal components, respectively. It is not known whether first metacarpal torsion is genetically or plastically developed. However, previous work on the foot has highlighted that the type of footwear people wear is associated with variations in metatarsal torsion observed in human populations (Drapeau and Harmon 2013). This suggests that torsion of the metatarsal could be at least partially caused by plastic deformation, and perhaps, this also happens to the first metacarpal. Although further study is needed, it is important to understand how this morphological trait of the first metacarpal varies, as this may alter the kinematics of the first CMC joint during functional motions.

It is important to mention the limitations to this study that may have influenced the findings. As cadaveric specimens were used in this work, it was not possible to obtain radiographic images that could be graded in the standard way for the presence of osteoarthritis. However, based on previous studies, symptomatic first CMC joint osteoarthritis is most prevalent in populations above 60 years of age (Niu et al. 2003; Wolf et al. 2014); the mean age of the specimens used in this study (53.5 ± 10.0 years) is below this. In addition, no osteophytes were observed on the CT scans, so the specimens were classified as healthy. Therefore, the variability of the anatomical features determined in this study cannot be directly associated with the development of first CMC joint osteoarthritis. Hence, further in vivo study with associated radiographic evidence is needed to determine how these anatomical features differ between healthy subjects and patients with first CMC joint osteoarthritis.

## Conclusion

Four anatomical features—articular tilt and torsion angles of the first metacarpal and length and width of the first metacarpal facet on the trapezium—were successfully measured on shape models obtained by performing multi-object SSM. The variability of these four anatomical features was associated with the first and second principal components of the model. Strong correlations were demonstrated between the anatomical features of the first metacarpal and trapezium. Further study is required to ascertain how these features affect the kinematics of the first CMC joint and how they may be linked with the formation or progression of first CMC joint osteoarthritis.
